# P-187. Strongyloides Seropositivity Among Patients Seeking Care at a Large Safety-Net Hospital System in New York City: Demographic Characteristics and Epidemiologic Trends

**DOI:** 10.1093/ofid/ofaf695.410

**Published:** 2026-01-11

**Authors:** Katrina Sandejas, Tamara Nawar, Jennifer Lee, Simona Bratu, John Quale

**Affiliations:** NYC Health + Hospitals/Kings County, Queens, NY; NYC Health + Hopitals/Kings County, Brooklyn, New York; NYC Health + Hospitals/Kings County, Queens, NY; NYC Health + Hospitals/Harlem, New York, New York; NYC Health + Hospitals/Kings County, Queens, NY

## Abstract

**Background:**

Strongyloidiasis affects over 600 million people globally and can persist asymptomatically for decades. Increasing global migration, particularly from endemic areas, has heightened the burden of neglected infections in the U.S. Since 2022, over 233,000 asylum seekers have arrived in New York City, straining public health systems, and coinciding with surges in communicable diseases such as tuberculosis. However, routine screening for parasitic infections like *Strongyloides* remains inconsistent.

**Methods:**

We conducted a retrospective analysis of *Strongyloides* serologic testing from January 2020 to December 2024 across 11 NYC Health + Hospitals facilities. For seropositive patients at four hospitals (that had increases in testing volume and positivity rates), chart reviews were performed to extract demographic data, immigration history, testing indications, and treatment information. Statistical analyses included t-tests and Chi square analyses.

**Results:**

Of 11,361 patients tested from 2020-2024 across the 11 hospital system, 1,914 (16.8%) were seropositive. Seropositive patients more likely to be older (57.0 vs. 53.2 years, P< .001), male (60.3% vs. 50.4%, P< .0001), and Spanish-speaking (48.6% vs. 35.7%, P< .0001) than seronegative patients. A marked increase in testing occurred at four hospitals. Testing rates at these four hospitals increased from 5.5 to 16.7 per 100000 hospital encounters (P< .0001), as did the number of seropositive cases (3.8 to 4.6 per 100000 encounters, P< .0001). A large number of migrant shelters were located near these four hospitals. Most patients originated from South America, Central America, or the Caribbean. Among 874 patients reviewed, eosinophilia (63.0%) and routine migrant screening (18.0%) were the most common testing reasons. Overall 13% did not receive therapy, highlighting treatment gaps.

**Conclusion:**

The surge in Strongyloides seropositivity, especially at hospitals near shelters, reflects the evolving impact of migration on public health. Targeted screening and presumptive treatment strategies are urgently needed. Allocating additional resources to disproportionately affected facilities is critical to managing the growing infectious disease burden among migrant populations.

**Disclosures:**

All Authors: No reported disclosures

